# Permittivity boosting by induced strain from local doping in titanates from first principles

**DOI:** 10.1038/s41598-023-30965-6

**Published:** 2023-03-07

**Authors:** Alex Kutana, Yuho Shimano, Ryoji Asahi

**Affiliations:** Nagoya University, Nagoya, Aichi 464-8603 Japan

**Keywords:** Materials science, Theory and computation

## Abstract

We examine the effect of isovalent substitutions and co-doping on the ionic dielectric constant of paraelectric titanates (perovskite, Ruddlesden-Popper phases, and rutile) using density functional perturbation theory. Substitutions increase the ionic dielectric constant of the prototype structures, and new dynamically stable structures with *ε*_ion_ ~ 10^2^–10^4^ are reported and analyzed. The boosting of ionic permittivity is attributed to local defect-induced strain, and maximum Ti–O bond length is proposed as a descriptor. The Ti–O phonon mode that is responsible for the large dielectric constant can be tuned by a local strain and symmetry lowering from substitutions. Our findings help explain the recently observed colossal permittivity in co-doped rutile, attributing its intrinsic permittivity boosting solely to the lattice polarization mechanism, without the need to invoke other mechanisms. Finally, we identify new perovskite- and rutile-based systems that can potentially display colossal permittivity.

## Introduction

Achieving materials with a very large (colossal) dielectric permittivity (CP) is crucial for various technological applications, including miniaturization of on-chip capacitive elements in transistors, high density memory, sensors, as well as high power density energy storage^1^. Due to their temperature and electric field stability, paraelectric materials are preferred for CP over ferroelectrics whose dielectric constant is high but also unstable near the phase transition temperature. With recent experimental reports on colossal permittivity in paraelectric TiO_2_ rutile via co-doping^2,3^, microscopic mechanisms for permittivity boosting need to be well understood. The apparent CP due to polarization of charge depletion layers at contacts, grain boundaries, interfaces, and other spatial heterogeneities^4,5^ (the so-called Maxwell–Wagner effect), is classified as “extrinsic”. Other extrinsic mechanisms include thermally activated conductivity by carrier hopping. A notable example is CaCu_3_Ti_4_O_12_, having a colossal permittivity close to 10^5^ by the Maxwell–Wagner effect^4,6–8^. On the other hand, possible bulk mechanisms including ionic polarization, metal–insulator transition, and charge density wave formation^8^ can be classified into “intrinsic”, i.e. bulk mechanisms that are active at all temperatures. Of these the ionic polarization is of greatest practical interest, as other mechanisms incur high dielectric losses, limiting applications. Since the early reports of large, temperature-dependent permittivity in insulating tetragonal YBa_2_Cu_3_O_6+*x*_^9,10^, the overlap of intrinsic and extrinsic processes has complicated pinpointing the microscopic origins of the apparent CP. In co-doped rutile, a thermally activated process, most likely the transport of hopping carriers, was found to be responsible for the largest part of the apparent colossal permittivity^3^. At the same time, a nonactivated, intrinsic mechanism of permittivity boosting coexists in Nb + In co-doped rutile. The mechanism does not switch off at low temperatures; in fact, the enhanced permittivity increases with decreasing temperature^3,11^. The temperature trend follows that of pure rutile, suggesting a common microscopic origin. While theory accounts well for the observed dielectric constant of pure rutile, and attributes it largely to the ionic mechanism, no theoretical explanation exists for permittivity *boosting* in co-doped rutile. Originally, the “electron-pinned defect-dipoles” mechanism has been proposed in In + Nb co-doped TiO_2_ rutile^2^, suggesting the electronic nature of the large response. Further studies of In + Ta and Al, Ga, In + Nb co-doped TiO_2_^12,13^ also attributed the observed colossal permittivity to the electronic mechanism. On the other hand, the studies of Nb + In co-doped TiO_2_ single crystals at sufficiently low temperature where thermally excited carriers are frozen suggested that the enhancement of the permittivity should stem from intrinsic effects^3,11^.

Here we show that the ionic mechanism can indeed account for boosting the permittivity in co-doped rutile and other substituted metal oxides. To this end, we perform a computational study of the ionic part of the dielectric permittivity of co-doped and substituted rutile and other Ti-containing dielectric oxides. We focus on substitutions in stable paraelectric prototypes, such as rutile TiO_2_, perovskite CaTiO_3_, and Ruddlesden–Popper Sr_2_TiO_4_ and and Sr_3_Ti_2_O_7_, modified via co-doping or isovalent replacement. From the analysis of the results, the permittivity increase in these materials is attributed to impurity-induced local strain, which enhances the dielectric response by softening the Ti–O phonon mode. We find that the frequency of the mode dominating the dielectric response can be changed over a wide range by substitutions, offering a possibility to precisely engineer the ionic dielectric constant.

## Results and discussion

We examine the effects of isovalent substitutions and co-doping for titanates on phonons, Born effective charges, and ionic part of the dielectric constant. The electronic part of the dielectric constant is typically ~ 1–10 across most insulators and was not considered here in detail. The prototypes for substitution include mineral perovskite CaTiO_3_ with Pnma structure, rutile TiO_2_, as well as Ruddlesden-Popper phases Sr_2_TiO_4_ and Sr_3_Ti_2_O_7_. Ca, Sr → Ba, Pb, Sr, Ca substitutions in perovskites, as well as III–V (Al, Ga, In, Sc, Y, La–V, Nb, Ta) and II-VI (Mg, Ca, Sr, Ba–Cr, Mo, W) co-doping in rutile are performed. A more detailed description of the parent compounds and substitutions is given in the Supplementary Information. A total of 150 rutile and 1178 perovskite and Ruddlesden-Popper structures were calculated. The goal is to analyze the structures where substitutions increase ε_ion_ and identify relevant phonon modes. Among dynamically stable titanates (ν^2^ > 0), we found the maximum Ti–O bond length, max(*d*_Ti-O_), reflecting maximum local strain, to be a good descriptor of the highest achievable ionic dielectric constant. The correlation between ε_ion_ and max(*d*_Ti-O_) is rationalized in terms of the softening of the phonon mode that corresponds to the Ti–O bond stretching. The descriptor was considered by analogy with Goldschmidt factor^14^, which is used as a descriptor for structural stability of perovskites. As high ionic dielectric constant is dominated by the Ti–O mode, lowering its frequency significantly increases the dielectric response.

The ionic part of the dielectric tensor ε_ion αβ_ is calculated as a sum over the gamma point phonon modes *s* according to^15,16^

1 $${\varepsilon }_{ion\,\alpha \beta }=\frac{{e}^{2}}{{{\varepsilon }_{0}M}_{0}V}{\sum }_{s }\frac{{\bar{Z}^*}_{s \alpha }{\bar{Z}^*}_{s \beta }}{({2\pi\nu }_{s })^{2}}$$

Here, *e* is an elementary charge, *M*_0_ is a convenient mass reference, typically taken to be 1 amu^16^, *V* a unit cell volume, ${\bar{Z}^*}_{s \alpha }={\sum }_{\kappa \beta }{Z^*}_{\kappa ,\alpha \beta }{\left({M}_{0}/{M}_{\kappa }\right)}^{1/2}{\xi }_{s ,\kappa \beta }$ is the *α*th Cartesian component of the unnormalized effective charge vector for phonon mode *s*, and ν_s_ is mode frequency. *ξ*_*s*,*κβ*_ are the eigenvectors of the dynamical matrix, normalized according to Σ_*κβ*_*ξ*_*s*,*κβ*_* ξ*_*s’*,*κβ*_ = δ_*ss’*_, and Z^*^_κ,*αβ*_ are the atomic Born effective charges, describing the proportionality^15,17^ between forces on atoms the and macroscopic electric field as ${eZ^*}_{\kappa ,\alpha \beta }=\partial {F}_{\kappa ,\beta }/\partial {\mathcal{E}}_{\alpha }$. Here, *F*_*κ*,*β*_ is the *β*th component of the force acting on the *κ*th atom, and ${\mathcal{E}}_{\alpha }$ is the *α*th component of the macroscopic electric field. As the mixed second derivatives of the total energy, Born effective charges can also be expressed as the derivatives of the components of the polarization vector ${\mathcal{P}}_{\alpha }$ with respect to the atomic displacements *τ*_*κ,β*_ as: ${eZ^*}_{\kappa ,\alpha \beta }=V\partial {\mathcal{P}}_{\alpha }/\partial {\tau }_{\kappa ,\beta }$. By analyzing the contributions to the ionic dielectric constant from the sum-over-modes Eq. (1), one can gain insights into the mechanisms of the dielectric constant boosting. Note that only modes with nonzero effective charges contribute to the static tensor Eq. (1). In order for the component of the mode effective charge $\bar{Z}^*$ to be large in any given direction, both the atomic Born effective charges Z^*^ and dynamical matrix eigenvector *ξ* need to have large components in that direction for the same atom.

**Figure 1:**
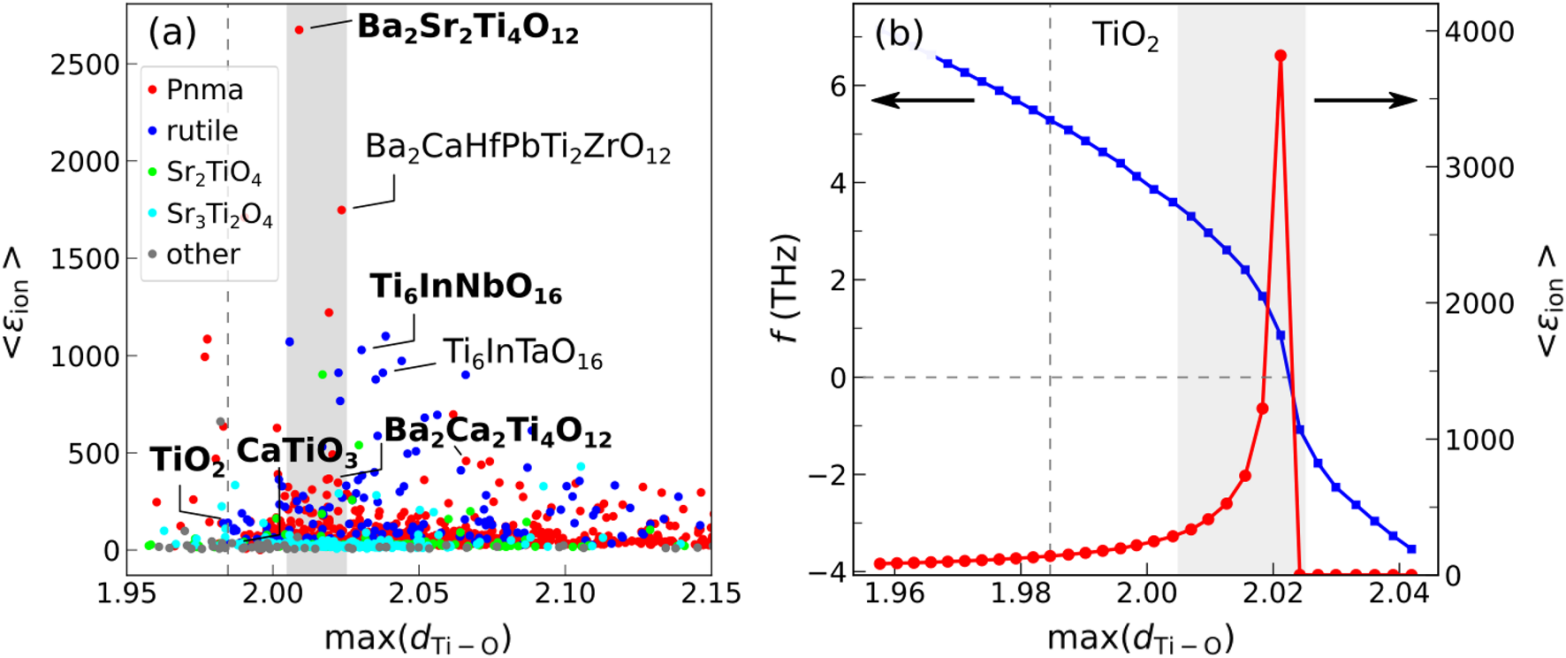
(**a**) Ionic part of the dielectric constant, *ε*_ion_, as a function of max(*d*_Ti–O_), the maximum length of the Ti–O bond in substituted/co-doped titanates. Different colors correspond to different prototype structures; (**b**) <ε_ion_> vs. max(*d*_Ti–O_) in rutile under uniform strain, showing a pole at the ferroelectric transition when the frequency of the A_2u_ mode becomes imaginary. The shaded area corresponds to the range of the maximum dielectric constants in (**a**); the vertical dashed line shows max(*d*_Ti–O_) at equilibrium lattice constant.

Figure 1a shows the dielectric constants in substituted/co-doped oxides as a function of max(*d*_Ti-O_), with a visible peak around max(*d*_Ti-O_) ≃ 2.02 Å. The average ionic dielectric constants of co-doped rutile TiO_2_ as a function of max(*d*_Ti-O_) is also shown in Fig. S1. Other geometric descriptors, such as the cationic radius of co-dopants, do not provide a good correlation with <ε_ion_>, as seen from Figs. S5 and S6. The value of max(*d*_Ti–O_) near the peak of ε_ion_ is higher than the typical bond lengths in titanates with moderate dielectric constants (e.g., in rutile, with the calculated <ε_ion_>  = 140, max(*d*_Ti–O_) = 1.98 Å), raising the possibility of mode softening by local strain from substitutions. The soft ferroelectric Ti–O mode has been studied extensively in titanates^18,19^, and its sensitivity to external perturbations such as volume changes or degree of Ti 3*d*–O 2*p* hybridization has been well recognized. In rutile, volume increase drives the Ti–O A_2u_ mode softening^20^, with frequency becoming imaginary and structure developing instability and possibly becoming ferroelectric at a certain extensive strain. The instability is manifested across different titanates^21^, and stabilized by anharmonic effects^22^. In Fig. 1b, we show the increase of <ε_ion_> with max(*d*_Ti–O_) in Ti rutile under uniform strain.The dielectric constant has a pole where the A_2u_ mode becomes unstable with its frequency becoming zero. The shaded area near the pole corresponds to the range of the maximum dielectric constants in Fig. 1a. It is seen that the maximum ε_ion_ in substituted/co-doped oxides is reached in the same range of max(*d*_Ti–O_) where the softening of Ti–O mode occurs in rutile. By examining the microscopic origin of the large response, it is indeed confirmed that the same soft Ti–O mode is responsible for the large dielectric constant in these oxides. Note that we do not find similar mode softening in Si- or Sn-based rutile structures, as seen from Figs. S2 and S3, where the dependencies of the dielectric constant on strain in TiO_2_, SiO_2_, and SnO_2_ rutile structures are compared.

**Figure 2:**
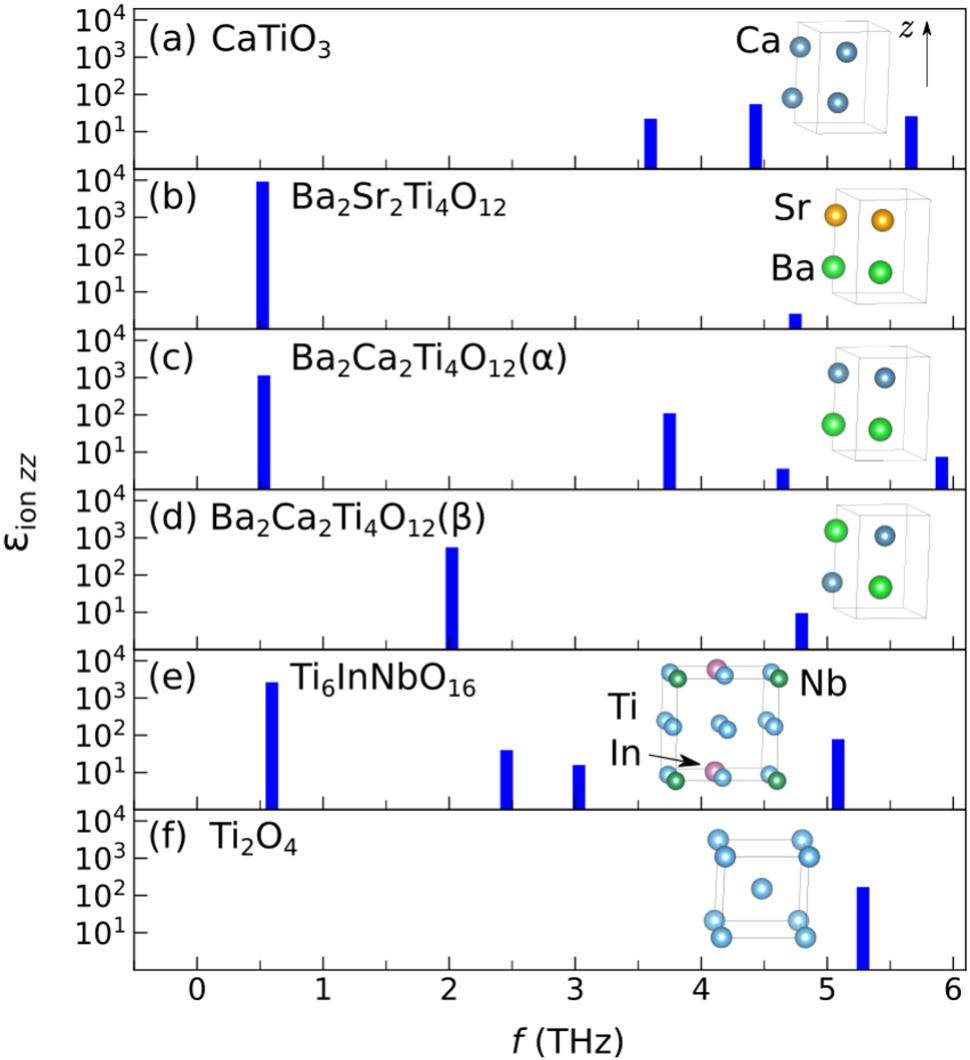
Phonon mode frequencies and their contributions to the sum over modes expression Eq. (1) for the ionic dielectric constant in substituted and co-doped titanates with a high *ε*_ion *zz*_ as well as the reference perovskite and rutile structures. Positions of Ca, Sr, Ba atoms and Ti, In, Nb in the unit cell are shown for perovskites and rutile, respectively, and O atoms are omitted. Dark blue—Ca, orange—Sr, green—Ba, blue—Ti, dark green—Nb, purple—In.

As an illustration, consider Ba_2_Sr_2_Ti_4_O_12_, obtained from the orthorhombic CaTiO_3_ by Ca → Ba, Sr substitutions, Fig. 2a and b. The structure has alternating planes of Ba and Sr normal to the *z* axis and is dynamically stable. It has an anisotropic ionic dielectric tensor with <ε_ion_>  = 3373, ε_ion *zz*_ = 9015, dominated by the lowest optical mode at ν_4_ = 0.521 THz, contributing 99.9% of ε_ion *zz*_. This is unlike CaTiO_3_, where many modes contribute to ε_ion_, as seen from the summary given in Table S1. In Ba_2_Sr_2_Ti_4_O_12_, max(*d*_Ti–O_) = 2.01 Å, similar to that of the dynamically unstable cubic BaTiO_3_, but unlike that structure, shorter Ti–O bonds are also present. Apparently, the ferroelectric Ti–O mode of BaTiO_3_ is stabilized by insertion of Sr planes, preventing the formation of unstable Ti–O chains. This effect can also be viewed as the softening of the infrared-active modes of CaTiO_3_ by Ca → Ba and Ca → Sr substitutions. Just like the rutile, the infrared-active modes of CaTiO_3_ are softened by tension^23^. Projecting this low energy mode onto the modes of cubic BaTiO_3_ shows that it is mostly made up of the ferroelectric Ti–O mode of BaTiO_3_, with a small contribution from the displacement of Ba against the Ti–O cage. Similarly, a large ε_ion *zz*_ = 1228 is achieved in a Ba_2_Ca_2_Ti_4_O_12_(α) structure with alternating Ba and Ca planes, Fig. 2c. Note that the frequencies of the dominant modes are similar in the Ba_2_Sr_2_Ti_4_O_12_ and Ba_2_Ca_2_Ti_4_O_12_(α) structures, and the larger ε_ion *zz*_ in Ba_2_Sr_2_Ti_4_O_12_ is due to its larger ${{\bar{Z}^*}_{4z}}$= 3.72, compared to ${{\bar{Z}^*}_{4z}}$= 1.33 in Ba_2_Ca_2_Ti_4_O_12_(α) in Fig. 2c. The dominant mode frequency is higher in another, Ba_2_Ca_2_Ti_4_O_12_(β), structure shown in Fig. 2d, ν_5_ = 2.02 THz, with ${{\bar{Z}^*}_{5z}}$= 3.52, giving ε_ion *zz*_ = 568 for that structure, as compared to ν_4_ = 0.53 THz, ${{\bar{Z}^*}_{4z}}$= 1.33 for the Ba_2_Ca_2_Ti_4_O_12_(α) in Fig. 2c. Thus, the frequency and effective charge of the dominant mode is sensitive not only to composition, but also to the atomic arrangement on the lattice at a fixed composition. By comparing the eigen displacements of the dominant modes ν_4_ and ν_5_ for the structures in Fig. 2c and d, respectively, the former has a significant motion of Ca and Ba planes against each other in the *z* direction, which is prevented in the latter by staggering Ca and Ba as in Fig. 2d. This staggered arrangement leads to dominant mode hardening from 0.53 to 2.02 THz.

We find this mechanism of permittivity enhancement through softening/mixing Ti–O modes to act in other substituted titanates. We obtain ε_ion *zz*_ = 252 in BaCa_3_Ti_4_O_12_ (not shown), a modest enhancement over the prototype CaTiO_3_ with ε_ion *zz*_ = 108. The dominant mode 5 in BaCa_3_Ti_4_O_12_ at 2.51 THz contributes ~ 184 to its *ε*_ion *zz*_. Projection onto the modes of *Pnma* BaTiO_3_ shows it to be a linear combination of the modes at 4.17*i* THz, 3.26 THz, and 5.26 THz of BaTiO_3_. The former is the ferroelectric Ti–O mode, whereas the latter two are Ba-only modes. Here, the stable A-site modes are mixed into the Ti–O mode, leading to its stabilization. Thus, lowering the symmetry of the structure via substitution is important for the stabilization of the Ti–O mode. This is to be expected, as the Ti–O chain structure instability is nonlocal, i.e., Ti and O atoms are coherently displaced along an infinite Ti–O chain, and there exists a minimum correlation length for the mode to become unstable^24^. The instability has been extensively analyzed^18,24^, with Ti 3*d*–O 2*p* hybridization and a partially covalent nature of the Ti–O bond being determined essential to the ferroelectricity^18,19^. The 1D chain-like nature of the instability was noted from the mode’s nearly flat zero-frequency isosurfaces in the Brillouin zone^25^. Real-space analysis has shown^24^ that the minimum length of the Ti–O chain for the instability to appear is ~ 10 atoms or 5 unit cells; thus any stabilizing perturbation must act on a shorter scale. In the Nb-In co-doped rutile, a soft mode with a large dielectric constant contribution appears at ν = 0.594 THz, as seen in Fig. 2e. In pure rutile, Fig. 2f, the active A_2u_ mode has a frequency of 5.28 THz.

**Figure 3:**
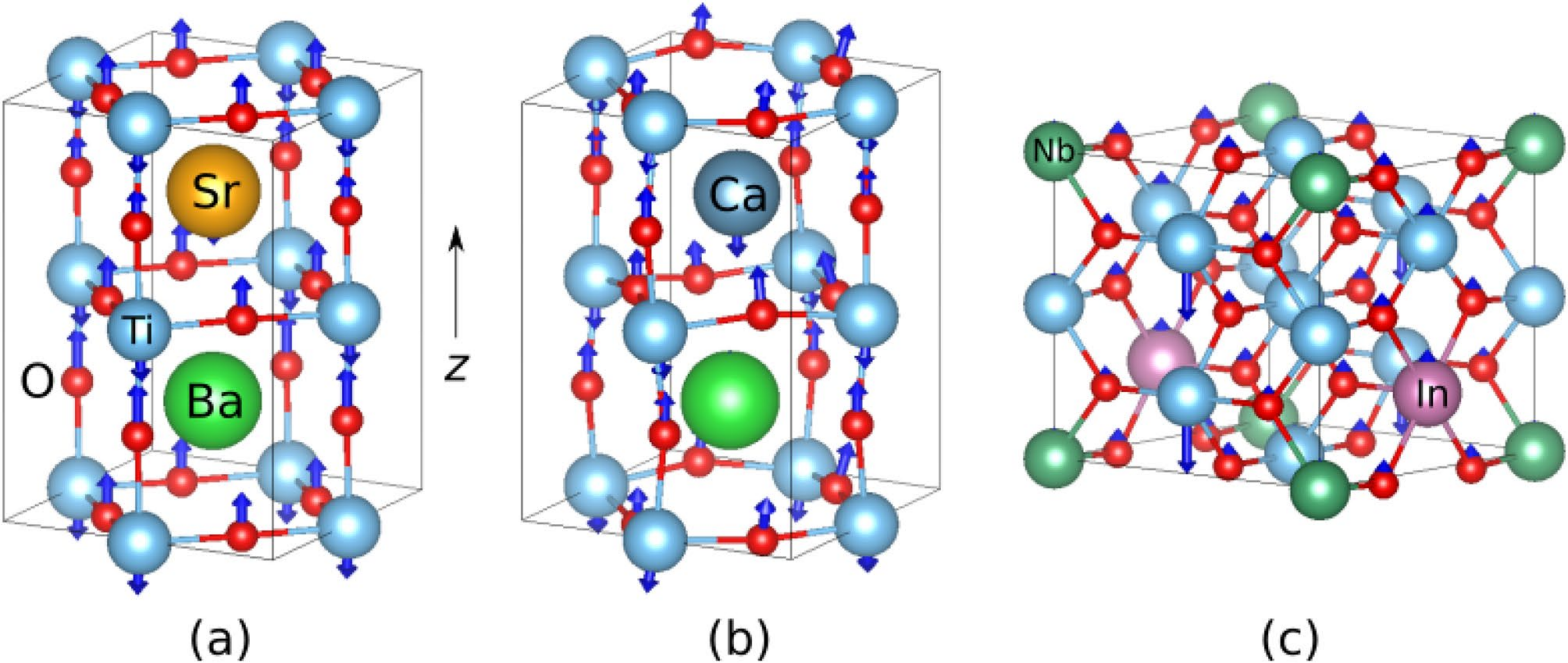
Eigen displacements ξ_*ν*,*κα*_/*M*_*κ*_^1/2^ of phonon modes relevant to the dielectric constant in substituted titanates (**a**) mode 4 in Ba_2_Sr_2_Ti_4_O_12_; (**b**) mode 5 in Ba_2_Ca_2_Ti_4_O_12_(β); (**c**) mode 4 in Nb-In co-doped TiO_2_ rutile. Blue—Ti, red—O, orange—Sr, dark blue—Ca, green—Ba, dark green—Nb, purple—In. For clarity, atoms outside of Ti–O cages are not shown in (**a**) and (**b**).

The visualizations of phonon modes responsible for the large dielectric response are shown in Fig. 3. In Ba_2_Sr_2_Ti_4_O_12_ and Ba_2_Ca_2_Ti_4_O_12_(α), shown in Fig. 3a and b, the atomic displacements are very similar to those of the ferroelectric BaTiO_3_ mode, which is also confirmed by mode projections. In contrast, in the Nb-In co-doped rutile, the mode is localized on a single Ti atom in the *xy* plane, and delocalized along the *z* direction, as seen in Fig. 3c, giving it a chain-like character, possibly due to symmetry breaking by co-doping.

We also note that the value ε_ion *zz*_ = 9015 obtained for one of Ba_2_Sr_2_Ti_4_O_12_ structures may be close to a practically achievable maximum value for the ionic dielectric constant. From Eq. (1), one has *ε*_ion_ ≃205 × (Z^*^/*ν*)^2^, assuming a cubic unit cell with a side of 6 Å, and mode frequency *ν* in THz. Thus, in order to have *ε*_ion_ ~ 10^4^, one needs Z^*^≃7 for *ν* = 1 THz. As larger Z^*^ values to achieve even higher *ε*_ion_ seem unrealistic, further increasing *ε*_ion_ then requires decreasing the mode frequency below 1 THz, which may eventually result in instability and switching to a ferroelectric state.

**Figure 4:**
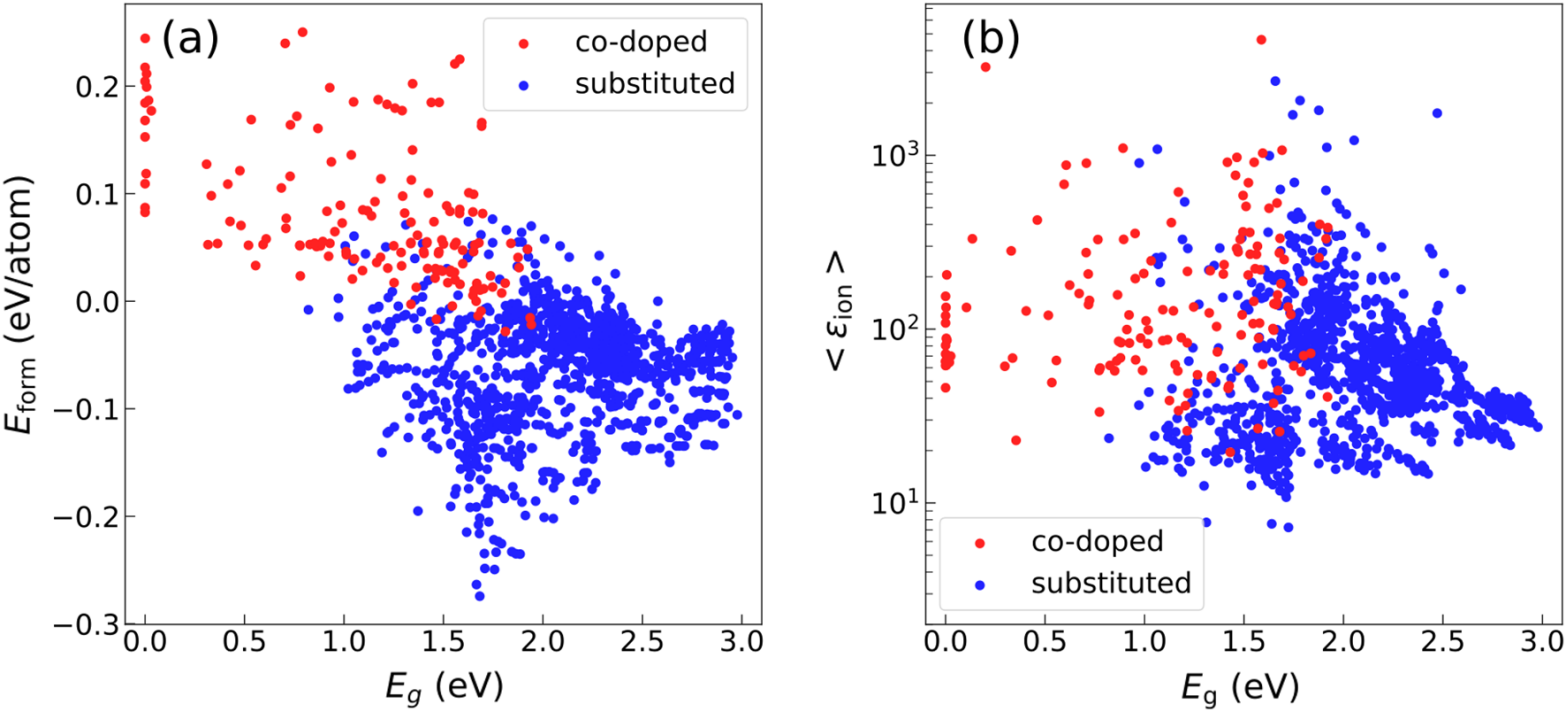
(**a**) Formation energies and (**b**) average ionic dielectric constants as a function of band gaps for Ti-containing oxides. Red—co-doped rutile, blue—substituted perovskite and Ruddlesden–Popper phases.

In order to assess the feasibility of synthesis of doped titanates, we calculate their formation energies by computing the energies of the reactions

$$\begin{aligned} & {\text{A}}_{x} {\text{B}}_{x} {\text{Ti}}_{{{2}({1} - x)}} {\text{O}}_{{2}} \to \, (x/{2}){\text{A}}_{{2}} {\text{O}}_{{3}} + \, \left( {x/{2}} \right){\text{B}}_{{2}} {\text{O}}_{{5}} + { 2}({1} - x){\text{TiO}}_{{2}} \\ & {\text{A}}_{x} {\text{B}}_{x} {\text{Ti}}_{{{2}({1} - x)}} {\text{O}}_{{2}} \to x{\text{AO }} + x{\text{BO}}_{{3}} + { 2}({1} - x){\text{TiO}}_{{2}} \\ \end{aligned}$$

for III–V and II–VI substitutions in co-doped rutile, respectively, and

$$\begin{aligned} & {\text{A}}_{x} A^{\prime}_{1 - x} {\text{TiO}}_{3} \to x{\text{AO }} + \, (1 - x)A^{\prime}{\text{O }} + {\text{ TiO}}_{2} \\ & A_{{\left( {n + 1} \right)x}} A^{\prime}_{(n + 1)(1 - x)} {\text{Ti}}_{n} {\text{O}}_{3n + 1} \to \left( {n + 1} \right)x{\text{AO }} + \, (n + 1)(1 - x)A^{\prime}{\text{O }} + n{\text{TiO}}_{2} ,\;\;n = 1, \, 2 \\ \end{aligned}$$

for the perovskites and Ruddlesden–Popper phases, respectively. The formation energy is defined as the energy difference per atom between the compounds on the left and right sides of the reaction. The obtained formation energies of titanates are shown in Fig. 4a, along with their band gaps. As can be seen from the figure, most of the substituted titanates have negative formation energies, favoring mixing, and moreover, have large band gaps. On the other hand, achieving co-doping in rutile could be more difficult, and some of the resulting compounds could be metallic. At the same time, there are a number of thermodynamically stable rutile compounds with moderate band gaps. Configurational energies, defined as the maximum formation energy difference among the structures of the same stoichiometry, are shown in Fig. S7. These can be rather small, e.g., ~ 3 meV/atom for Ba_2_Sr_2_Ti_4_O_12_, compared with its formation energy of ~ − 145 meV/atom. The closeness in energy among different configurations indicates that their mixing should occur at finite temperatures, and thermodynamic averaging, together with consideration of anharmonic effects, should be performed in order to estimate the dielectric constant at nonzero temperature.

CP materials must possess sufficiently large band gaps in order to prevent dielectric breakdown, which is believed to be electronic in nature^26^. Our calculated average ionic dielectric constants as a function of the band gap are shown in Fig. 4b, and the electronic contribution to the dielectric constant is shown in Fig. S4. Note that the electronic contribution does not exceed 10 for materials with band gaps > 0.6 eV. It is seen that generally insulators with larger dielectric constants tend to have smaller band gaps. This undesirable trend is consistent with the empirically known inverse relation between the dielectric constant and dielectric breakdown strength^27^. Discovering CP materials with both high dielectric constant and wide band gap remains a challenge that must be addressed in the future work.

Based on the results of first-principles calculations, we show that co-doping and substitutions offer a versatile and efficient pathway to increase the ionic dielectric constant in titanate paraelectrics. Most of the obtained compounds are found to be both dynamically and thermodynamically stable, offering a good prospect of experimental synthesis. Large ionic dielectric constants *ε*_ion_ ~ 10^4^ can be achieved in some compounds, establishing them as new candidates for colossal permittivity materials. The microscopic origin of the permittivity increase, namely the softening of the optically active phonon mode by a local defect-induced strain, may point to a new direction to search for colossal permittivity in other materials.

## Methods

The ionic dielectric response was calculated using density functional perturbation theory (DFPT) approach, as implemented in the VASP package. PBEsol functional^28^ with Hubbard *U* correction was used. *U* = 3 eV was applied to *d* electrons in transition metals, except for Ti and Sc, where *U* = 0 was used. The full list of the *U* values is given in the Supplementary Information. The k point grid density of 3000 k points·atom (approximately corresponding to a distance of ~ 0.2 Å^−1^ between the k points) was used for the integration over the Brillouin zone. Full cell shape and atomic position relaxations were carried out prior to DFPT calculations.

## Supplementary Information

Supplementary Information.

## Data Availability

The datasets generated during the current study will be available at https://doi.org/10.17632/n9j8h4h9g3.1 following a 6 month embargo from the date of publication to allow for patenting of research findings.
