# Supplementary material for: Permittivity boosting by induced strain from local doping in titanates from first principles
Source: Sci Rep. 2023 Mar 7;13:3761. doi: 10.1038/s41598-023-30965-6 (PMC9992386; doi:10.1038/s41598-023-30965-6)
Supplement: Supplementary file 1 — Supplementary Information. [file 41598_2023_30965_MOESM1_ESM.pdf]

Supplementary Information for Permittivity Boosting by Induced Strain from Local Doping in Titanates from First Principles

Alex Kutana, Yuho Shimano, Ryoji Asahi
Nagoya University, Nagoya, Aichi 464-8603, Japan

The parent compound for substituted perovskites is a paraelectric calcium titanate $CaTiO_3$ (mineral perovskite) that crystallizes into an orthorhombic *Pnma* (62) phase at low temperatures;[1] the observed high temperature phases include tetragonal *I*4/*mcm* (140), and a cubic $Pm\underline{3}m$ (221) phase for T>1580 K.[2,3] The breaking of the ideal cubic $Pm\underline{3}m$ symmetry occurs by $a^-a^-c^+$ tilting of the TiO_6 octahedra. Our calculated average ionic dielectric constant for this material at PBEsol lattice constant is 124, in the range of previously reported theoretical values[4] ~93-268, which varied depending on whether a theoretical (LDA) or experimental value of the lattice constant was used. The Ruddlesden-Popper phases[5,6] Sr_2TiO_4 and $Sr_3Ti_2O_7$ have the I4/mmm (139) symmetry, and TiO_2 rutile has the $P4_2$/mnm (136) symmetry.

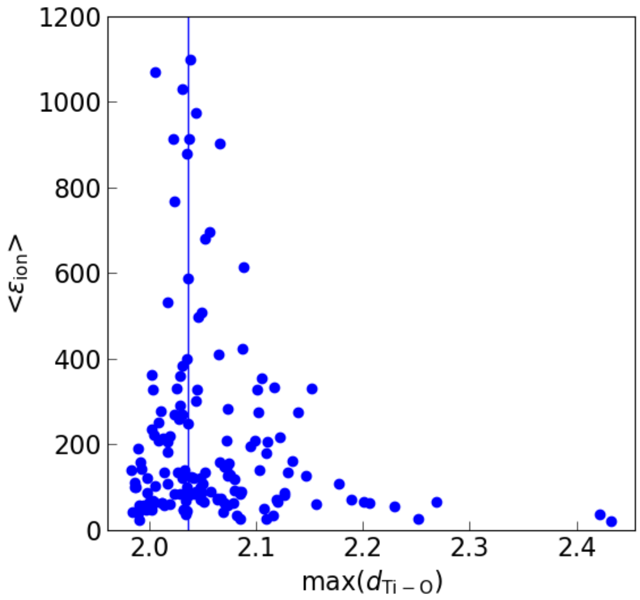

Figure S1. Ionic part of the dielectric constant, ε_{ion}, as a function of max($d_{Ti\text{-}O}$), the maximum length of the Ti-O bond in co-doped TiO_2 rutile. The vertical line at max($d_{Ti\text{–}O}$)=2.036 Å is drawn to guide the eye.

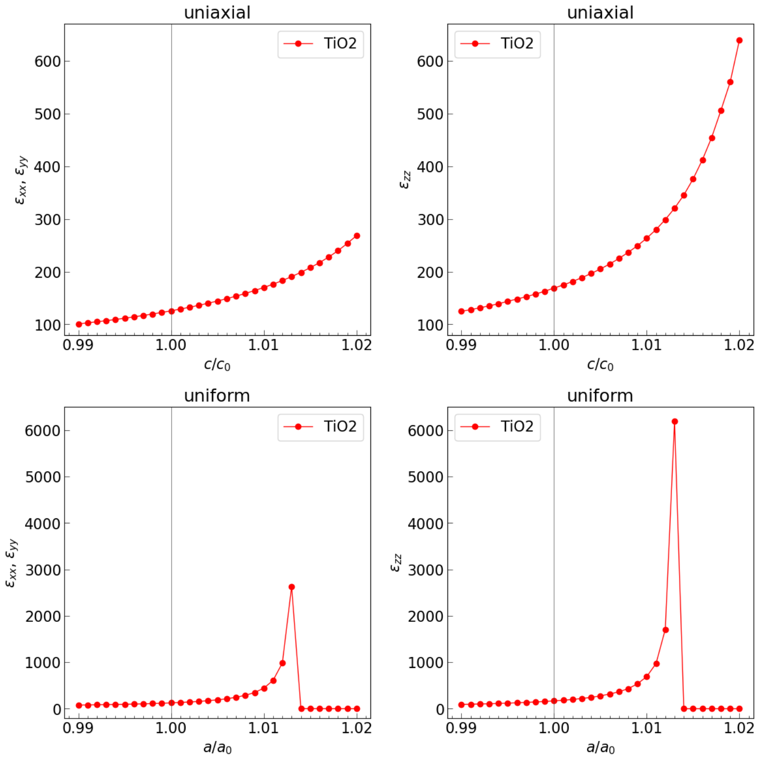

Figure S2. ε_{ion} of TiO_2 rutile as a function of uniaxial and uniform strain.

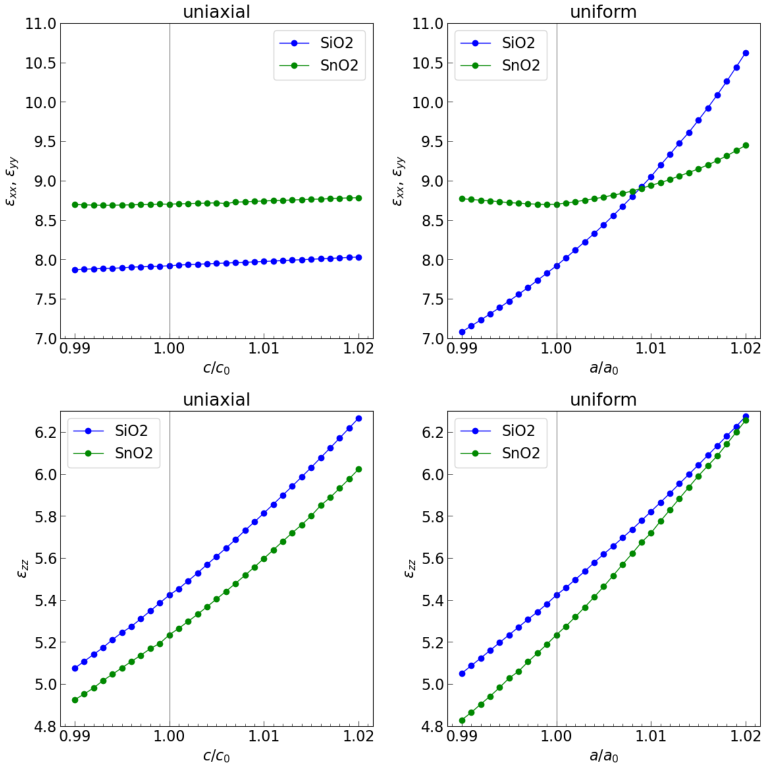

Figure S3. ε_{ion} of SiO_2, SnO_2 rutile structures as a function of uniaxial and uniform strain.

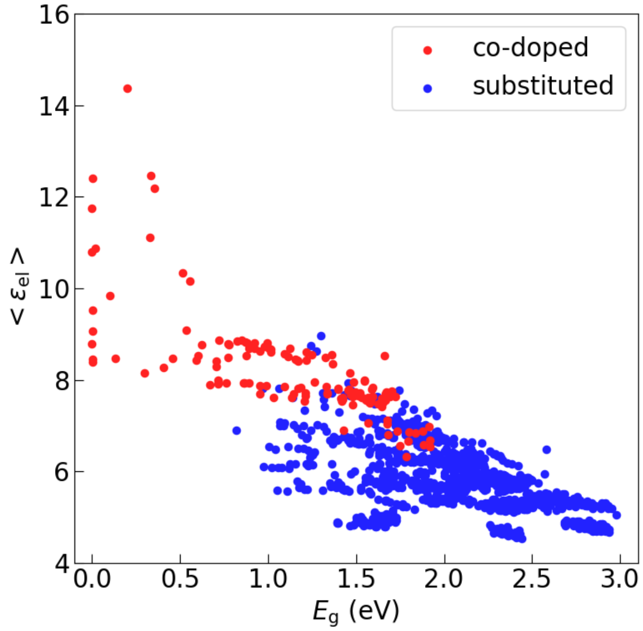

Figure S4. Electronic contribution to the dielectric constant <εel> as a function of the band gap in Ti-containing oxides. Red - co-doped rutile, blue - substituted perovskite and Ruddlesden-Popper phases.

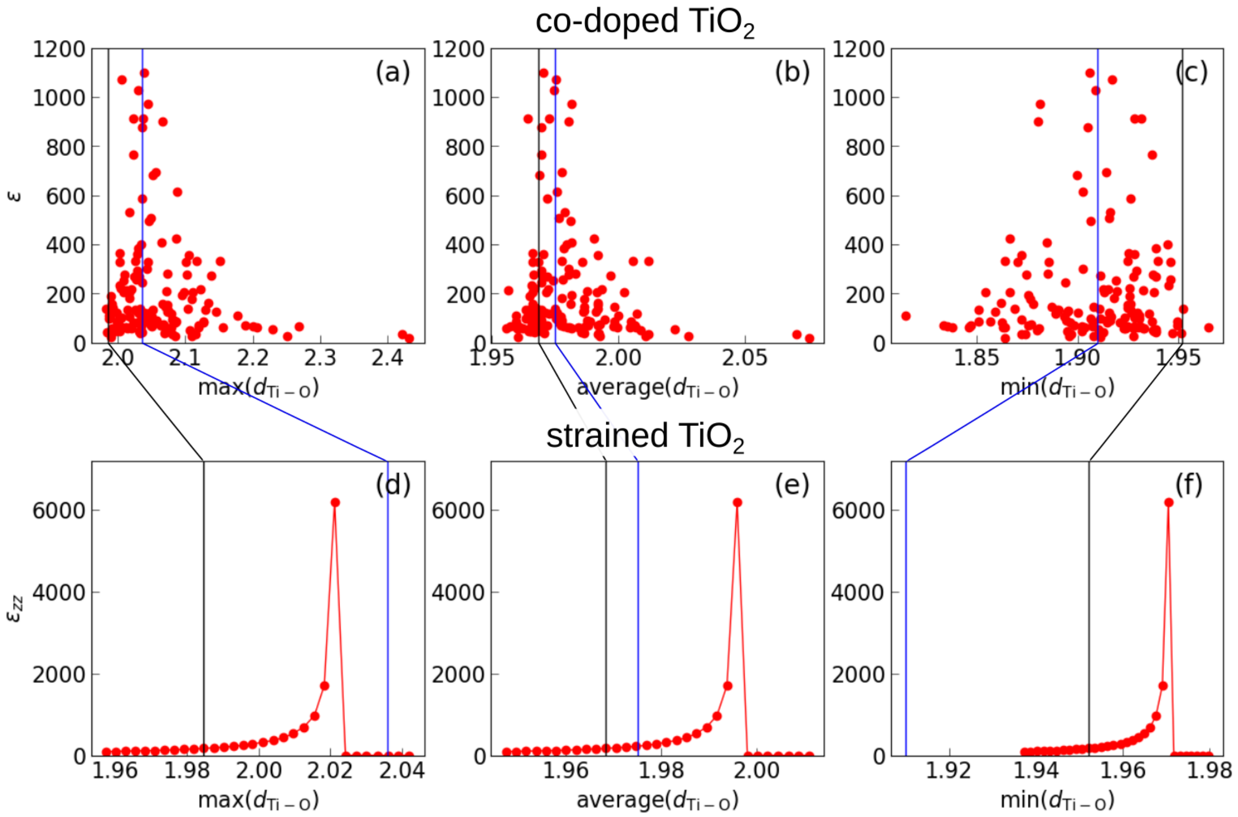

Figure S5. Ionic dielectric constant in (top row) co-doped rutile TiO_2 vs. (a) maximum, (b) average, (c) minimum Ti-O bond length, and (bottom row) uniformly strained rutile TiO_2 vs. (d) maximum, (e) average, (f) minimum Ti-O bond length. Vertical black lines show bond lengths in unstrained rutile, and vertical blue lines are at the positions where maximum dielectric constant is achieved in co-doped rutiles.

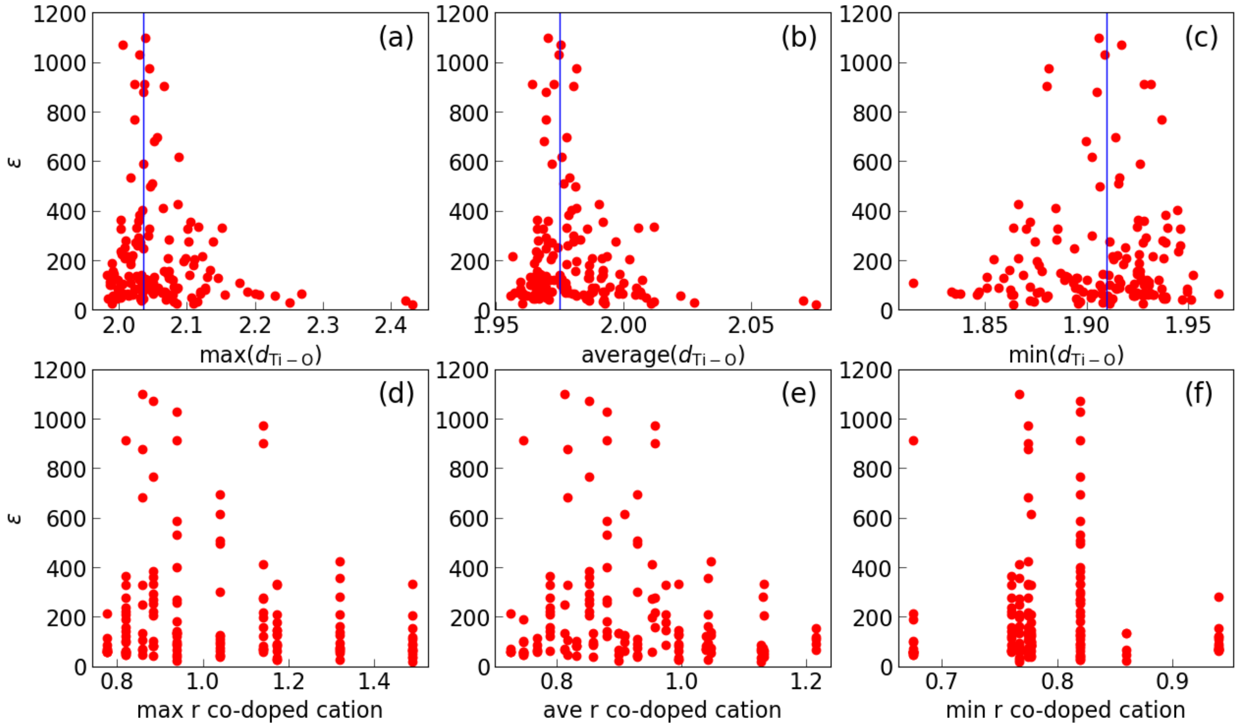

Figure S6. Ionic dielectric constant in co-doped rutile TiO_2 vs. (a) maximum, (b) average, (c) minimum Ti-O bond length, and (d) maximum (e) average, (f) minimum cationic radius of co-dopants. The cationic radii from the pymatgen package[7] were used.

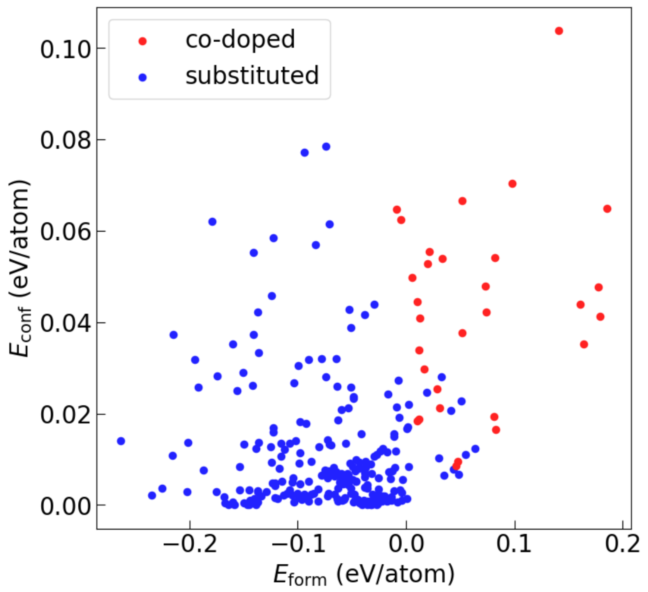

Figure S7. Configurational energies of Ti-containing oxides. Configurational energy is defined as the maximum formation energy difference among structures of the same stoichiometry.

Table S1. Phonon modes of Pnma $CaTiO_3$ contributing to dielectric constant.

	ν (THz)	$\varepsilon_{partial}$	mode #
εxx			
	3.828	109.1	6
	5.026	19.9	12
	6.379	2.5	21
εyy			
	4.021	96	7
	5.222	24.1	14
	9.172	2.6	31
	15.56	2.2	53

εzz			
	3.599	22.4	5
	4.432	55.0	9
	5.668	26.3	16
	15.90	2.5	55

Table S2. Ionic part of the dielectric tensor in substituted and co-doped titanates shown in Fig. 2.

compound	ε_{xx}	ε_{yy}	ε_{zz}
$CaTiO_3$	136.0	126.9	108.2
$Ba_2Sr_2Ti_4O_{12}$	697.0	407.2	9015.2
$Ba_2Ca_2Ti_4O_{12}$ (α)	92.8	53.4	1228.7
$Ba_2Ca_2Ti_4O_{12}$ (β)	105.1	432.5	564.2
$BaCa_3Ti_4O_{12}$	175.4	186.3	252.4
Ti_6InNbO_{16}	213.4	106.6	2767.9
Ti_2O_4	125.8	125.8	169.0

Table S3. Born effective charges $Z^*_{\kappa,zz}$ in $Ba_xSr_yCa_{1-x-y}TiO_3$. O_1 atoms are part of Ti-O chains aligned with the z axis, O_2 atoms are part of other Ti-O chains. For symmetry inequivalent atoms in substituted structures, maximum value is taken.

	Ca	Sr	Ba	Ti	O_1	O_2
$CaTiO_3$	2.38	–	–	7.32	-5.76	-1.97

$Ba_2Sr_2Ti_4O_{12}$	–	2.54	2.7	7.69	-6.35	-2.09
$Ba_2Ca_2Ti_4O_{12}$ (α)	2.51	–	2.62	7.30	-6.12	-2.06
$Ba_2Ca_2Ti_4O_{12}$ (β)	2.51	–	2.61	7.57	-6.16	-2.11
$BaCa_3Ti_4O_{12}$	2.46	–	2.66	7.41	-6.14	-2.07
$BaTiO_3$	–	–	2.72	7.74	-6.16	-2.15

Table S4. U values used for Hubbard correction applied to *d* electrons.

el	O	Mg	Al	Ca	Sc	Ti	V	Cr	Ga	Sr	Y	Zr	Nb	Mo	In	Ba
U (eV)	0	0	0	0	0	0	3	3	0	0	3	3	3	3	0	0

el	La	Hf	Ta	W	Pb
U (eV)	3	3	3	3	0

The atomic structures and calculated properties are provided in json format.

References

(1) Kay, H. F.; Bailey, P. C. Structure and Properties of CaTiO3. *Acta Crystallogr.* **1957**, *10* (3), 219–226. https://doi.org/10.1107/S0365110X57000675.
(2) Kennedy, B. J.; Howard, C. J.; Chakoumakos, B. C. Phase Transitions in Perovskite at Elevated Temperatures - a Powder Neutron Diffraction Study. *J. Phys. Condens. Matter* **1999**, *11* (6), 1479–1488. https://doi.org/10.1088/0953-8984/11/6/012.
(3) Yashima, M.; Ali, R. Structural Phase Transition and Octahedral Tilting in the Calcium Titanate Perovskite CaTiO3. *Solid State Ion.* **2009**, *180* (2), 120–126. https://doi.org/10.1016/j.ssi.2008.11.019.
(4) Cockayne, E.; Burton, B. P. Phonons and Static Dielectric Constant in CaTiO3 from First Principles. *Phys Rev B* **2000**, *62* (6), 3735–3743. https://doi.org/10.1103/PhysRevB.62.3735.
(5) Ruddlesden, S. N.; Popper, P. New Compounds of the K_2NiF_4 Type. *Acta Crystallogr.* **1957**, *10* (8), 538–539. https://doi.org/10.1107/S0365110X57001929.
(6) Ruddlesden, S. N.; Popper, P. The Compound $Sr_3Ti_2O_7$ and Its Structure. *Acta Crystallogr.* **1958**, *11* (1), 54–55. https://doi.org/10.1107/S0365110X58000128.
(7) Ong, S. P.; Richards, W. D.; Jain, A.; Hautier, G.; Kocher, M.; Cholia, S.; Gunter, D.; Chevrier, V. L.; Persson, K. A.; Ceder, G. Python Materials Genomics (Pymatgen): A Robust, Open-Source Python Library for Materials Analysis. *Comput. Mater. Sci.* **2013**, *68*, 314–319. https://doi.org/10.1016/j.commatsci.2012.10.028.
